# 1896. Analysis of Virulence Factors and Antibiotic Resistance Genes in *Mycobacterium abscessus* Prophage Sequences genomes and Relationships with Clinical Patient Prognoses

**DOI:** 10.1093/ofid/ofad500.1724

**Published:** 2023-11-27

**Authors:** Luan Sichun

**Affiliations:** Zhongshan Hospital, Fudan University, Shanghai, Shanghai, China

## Abstract

**Background:**

*M. abscessus* is known for its multiple resistance to antibiotics, and diseases caused by *M. abscessus* have limited methods to treat with. Little is known for prophages carried by *M. abscessus*, nor their role played in diseases.

**Methods:**

Strains identified as *M. abscessus* from patients from December 2017 to August 2021 were included. Prophages, virulence factors and antibiotic resistance genes in genomes were analyzed. The correlation between prophages, virulence factors and antibiotic resistance genes contained, and clinical patient prognoses were analyzed. The sequence similarity between prophages and known phages were analyzed.

**Results:**

A total of 145 prophage sequences were detected in 56 *M. abscessus* strains. The average number of intact prophages was 0.7 ± 0.6. The average number and gene proportion of virulence factors and drug resistance genes in prophages were higher than those in known mycobacteriophages. Differences of average prophage carried, virulence factors and drug-resistant genes, and their proportions between different prognoses were not statistically significant, except for virulence factors. The average similarity of prophage sequences in single patient was 99.86%; which was significantly higher of the average matching rate of 89.27% in different patients and the total similarity of 78.28% between prophages and known phages. No clear positive correlation between tested common virulence factors or virulence factors and clinical patient prognoses was observed.

**Conclusion:**

*M. abscessus* commonly carries prophages, and they have higher contents of virulence factors and drug resistance genes than known phages. The prophage detected in patients with good prognosis carried more virulence factors, and its meaning was unclear. The composition of the prophage sequences in the strains isolated from the same patient is stable, with occasional base mutation or deletion. Prophages carried in strains of different patients were highly diverse, and shared low similarity with known mycobacterium phages.

**Disclosures:**

**All Authors**: No reported disclosures

